# Dissecting systemic racism: policies, practices and epistemologies creating racialized systems of care for Indigenous peoples

**DOI:** 10.1186/s12939-021-01500-8

**Published:** 2021-07-14

**Authors:** Sarah Louise Fraser, Dominique Gaulin, William Daibhid Fraser

**Affiliations:** 1 School of Psychoeducation, University of Montreal, Pavillon Marie-Victorin, C. P. 6128, succursale Centre-ville, Montréal, QC H3C 3J7 Canada; 2 Centre de Recherche en Sante Publique (CReSP), University of Montreal and CIUSSS du Centre Sud-de-L’Île de Montreal, Montreal, Canada; 3 School of Social Work, University of Montreal, Montreal, Canada

**Keywords:** Systemic racism, Epistemic racism, Indigenous health, Indigenous knowledge

## Abstract

In this paper we explore some of the ways systemic racism operates and is maintained within our health and social services. We look at a very specific context, that of Nunavik Quebec, land and home to 13,000 *Nunavimmiut*, citizens of Quebec and Canada, signatories of the James Bay and Northern Quebec Agreement. We operationalize some of the ways in which policies and practices create and support social hierarchies of knowledges, also called epistemic racism, and how it impacts our ability to offer quality care that Indigenous peoples can trust and use.

## Background

Recently in Quebec, the death of Joyce Echaquan sparked outrage and has led to the community-based movement *Justice for Joyce*. Atikamekw woman and mother of 7, Joyce recorded her nurses speaking to her in an overtly racist fashion a few hours before her death. A few months later, a homeless Innu man died on a cold January night after having to leave a shelter that had to close its doors in the evening due to policies developed during the Covid pandemic. In the wake of these tragic and preventable deaths, Indigenous peoples of Quebec are speaking publicly, as they have been for years. The outcries speak not only of interpersonal racism but also of systemic racism within our systems of care and the need to address these structural realities that impact the health and wellbeing of Indigenous peoples [1]. Yet, provincial government representatives were and to this day continue to be unable to speak to the presence of systemic racism, and instead focus on the idea that most people working in health care are not racist individuals. These statements suggest a misunderstanding of the concept and underlying mechanisms of systemic racism. The Commission des Droits de la Personne et de la Jeunesse [2] defines systemic racism `as the sum of disproportionate exclusionary effects resulting from the combined effect of prejudice and stereotypical attitudes, often unconscious, and policies and practices generally adopted without taking into account the characteristics of members of groups covered by the prohibition of discrimination. As non-Indigenous researchers in the field of Indigenous health and social services, more specifically with Inuit of Nunavik, Quebec, we wondered how we could shed light some on of the structural dimensions that allow systemic racism to take place within the institutions of health and social services. We start by describing the history of health and social services for Inuit in northern Canada. Then we offer a short description of the nature of our research in Nunavik, Quebec. From the research findings, we describe certain realities that influence the experience of Inuit workers within the services, and family members using the services. These realities suggest the presence of epistemic racism [3, 4]: the hierarchy and segregation of knowledges which ultimately influences who makes clinical decisions, how the care is offered and how services are funded. Epistemic racism, as will be illustrated below, is a root cause of systemic racism. We end with lessons learned from promising movements in Nunavik that may allow for slow shifts towards cultural safety and ultimately self-determination of care for families and communities.

## Main text

### Colonial history in northern Quebec

Nunavik is the northernmost region of Quebec, Canada, and home to approximately 13,000 Inuit living in 14 isolated communities, with populations varying between 300 and 3000. Communities can be accessed by plane or boat.

Prior to contact with European settlers, health and social issues were dealt with by kin, with people being recognized for their knowledge and gifts in supporting others in their health and wellness [5, 6]. In the 1950’s, with the arrival of icebreakers meant to detect and treat people with tuberculosis, the medical and social fields were rapidly taken-over by external forces including the government and the church [7]. Later, in the 70 s with the signing of the James Bay Agreement came a clear request that services be governed by Inuit. Inuit (and Cree) agreed to open their territories to hydroelectric developments and all other forms of exploitations of natural resources [7]. In return, the province recognized certain specific rights to Cree and Inuit. School boards and health services were created, and a regional government was established through this Agreement [8].

The current education and health institutions can be led by an Inuk executive director with an Inuit Board of Directors, however, the institutions remain dependent on provincial government funding and have limited forms of decision-making power regarding funding and policy.

It is in this political context that our research team was invited to work with community and regional leaders of Nunavik in a series of interrelated research projects which aim to better understand the availability, and quality of services and resources in place for and by Inuit families as they look at ways to enhance cultural safety of the services within the region. As part of this research program, we created a community advisory board, held community workshops, then conducted 14 interviews with community members to understand their experiences with services and their needs for family wellbeing [9]. A qualitative project was also conducted to map out existing services within the region. A total of 60 interviews were conducted with Inuit and non-Inuit individuals, working for either a school, youth protection services, daycare, police, nursing station, hospital, health board, community committee, or, who represented families using services [10].

Later, we interviewed 19 key informants from 5 communities around Nunavik to better understand conceptualizations of wellbeing and Inuit-led practices that support wellness [11]. These various projects were meant to support community actions [12] and regionally led services [13].

When juxtaposing the findings of the various projects, we came to observe points of tensions and incongruities that indicated the clear presence of certain factors that synergistically participated in creating systemic racism. In the following sections we briefly describe certain issues that come to light and point towards a mechanism by which systemic racism takes place: the creation of hierarchies of knowledge and the segregation of these knowledges also known as epistemic racism.

### Inuit, not present in clinical decision-making

Until approximately 2012, Inuit could take on certain positions as clinical workers in the social realms without having a Bachelor’s degree in these fields. However, in 2012 changes in policy and regulations in Quebec led to a situation where certain clinical acts could only be conducted by people who are members of professional orders and had completed specific degrees (social work, psychoeducation, psychology). At the current time there is no post-secondary education available in Nunavik. To achieve the required credentials, Inuit must move to urban areas in southern Quebec for several years on a full time basis. Inuit are usually hired as secretaries, interpreters and community workers. Their roles are often limited to translation, interpretation, cultural mediation, and being in training with non-Inuit workers [14]. Thus, they are generally excluded from clinical discussions as they are perceived by many non-Inuit workers as not having the training nor the affiliations to professional orders that would allow them to partake in confidential clinical case discussions [14].

Paradoxically, in the social and youth protection fields in Northern Quebec, the difficulty of recruiting people who have these degrees has led to the recruitment of non-Inuit workers who are currently studying or who have completed a college or bachelor level program in a connected field without having the credentials to be a member of an order. Many non-Inuit workers hold positions or take on duties that are much more specialized than what they would be allowed to hold in Southern Quebec.

Moreover, due to lack of housing and challenges of recruiting staff, more and more non-Inuit workers, especially coordinators, directors and administrators are doing tele-work from southern Quebec, implying that certain clinical decisions related to health and social issues are being made outside of the communities [10].

### Inuit knowledge not seen as being part of the health system

Not only are Inuit less present around clinical decision-making; Inuit conceptualizations of wellness and wellness practices are relegated to the realm of community. Discussions around clinical vignettes, as well as around broader questions related to community wellness and family wellbeing allowed us to document Inuit practices and approaches related to children and families [13]. Responses were similar from community to community and across studies. People had a clear understanding of approaches and practices that are supportive in the prevention and intervention of social and mental health challenges that youth and families experience [13]. However, when attempting to transmit knowledge that had been shared to us by Inuit workers and families, certain non-Inuit workers would say things such as: yes, it is interesting, but those are *community activities*, they aren’t part of prevention or intervention, this isn’t health. This catch-22 is typical of a division between culture and health, community and services. This division is not desired by community members but is instead imposed by the structures and policies that regulate the practices within health and social institutions.

### What happens when neither Inuit workers nor cultural and community knowledge are integrated within services?

Since many Inuit find it difficult to have a place within the services, and that these services do not necessarily reflect their values, knowledge and ways of interacting, many Inuit choose not to go to the services until a crisis erupts. Inuit families talk about their fear of seeking help, fear of being judged or misunderstood by workers regarding their educational methods and fear that their child will be taken into the care of child welfare [15]. Families may try to use the services but may terminate their follow-up early which can at times lead to the imposition of services through court orders [16]. Non-Inuit workers sometimes understand these abrupt terminations as Inuit being resistant to the help offered, or as Inuit `culture` not being prevention-oriented [16].

### Communities are invited to mobilize to offer culturally adapted services with limited support

As mentioned above, Inuit have a clear picture of what is needed in order to be well as individuals, families and communities. Key actors in Nunavik have developed projects and programs for their communities, sometimes autonomously, sometimes with external support. Activities include on the land camps, cultural events, youth forums, community kitchens, family houses and more. Community spaces often provide the bridge between community needs and institutional services in a manner in which community members are included. However, often relegated to the community-level, these projects are constantly in need of funding. Community members that initiate projects for their community, or who start community organizations to offer community services must complete funding requests and administrative documentation to ensure that services can live-on the following year [17]. Workers within community-led organisations receive smaller salaries than those that are offered within institutions, with very little benefits. The realities of community workers are complex. Community workers often lack psychosocial and organizational support in order to offer consistency in their services [17]. The perennity of services is precarious. Northern organizations administered by Inuit, including the Nunavik Regional Health Board, have been working hard to find ways to continuously support and fund these community initiatives, however the resources for these out-of-institution services are always limited, in part due to the fact that funding is recurrent and funds for prevention-oriented public health care are very limited. Moreover, governmental funding sent to institutions and to communities is generally attached with predefined projects or programs that are ill-adapted to the realities of the community. The onus of adapting is on the institutional agents or community members of the region that already have other plans in place.

These realities create a racialized two-tracked system with unequal funding and a constant need to re-adapt, re-adjust, re-apply to available funding rather than invest in the plans being created by communities and regional institutions.

#### Dichotomization of knowledge: a social, political and economic construct. Not a scientific one

The points described above highlight the limiting impact that policy has on Inuit agency in institutional services, including the possibilities for Inuit to work within the systems of care and to influence the nature of the services that are offered to Inuit families. Directly related to these policies and practices is the dichotomization of forms of knowledges: Clinical versus Cultural, Health versus Community. Clinical knowledge is understood as belonging to institutions. The dichotomization is a political and economic reality built on social constructs of whom has knowledge and which knowledge is valuable to health.

Yet research in the field of transcultural psychiatry clearly demonstrates that in reality, clinical and cultural are highly interrelated in a variety of ways [18, 19]. Clinical diagnoses and intervention plans are socio-cultural scripts [19–21]. The multiple transformations of the guide book for diagnosing mental illness, the Diagnostic and Statistical Manual of Mental Disorders, over the past 50 years is an obvious demonstration of the constant metamorphosis of professional understanding and description of mental health and wellness [21, 22]. Studies conducted with people from different cultural backgrounds demonstrate how diagnoses and interventions depend on culture, and how culture is embedded in every aspect of mental health and wellbeing [23, 24]. Culture influences when, where, how, and to whom individuals tell their experiences of illness and distress [23], the patterning of symptoms [25], and the models clinicians use to interpret and understand symptoms [24, 25]. Culture also influences peoples’ perceptions of how services should be provided, by whom, and in which form.

Culture is also clinical in the sense that culture can be a form of psychosocial intervention [18, 24, 26]. Cultural activities can act as a buffer for mental health difficulties, and many publications have demonstrated the effectiveness and modalities of actions of interventions that are based on traditional knowledge and land-based activities [18, 25, 26]. Any attempt to segregate culture from clinical or to relegate culture to local people and clinical to institutional professionals is not only a clinical mistake but a perpetuation of colonialism and of privilege for certain social groups. By segregating the cultural from the clinical, we perpetuate the hierarchy of knowledges: epistemic racism.

#### Moving forward

There are important initiatives that are taking place and clearly identifying what is needed for change. The Sukait Steering Committee was created in 2017 to help guide the transformation of integrated services for children, youth and families. Inuit suicide prevention liaison officers throughout Nunavik are working together to develop best practices to support community efforts in suicide prevention. To this effect, a yearly conference called Puttautit is designed and led by Inuit. Family houses are being created in communities across Nunavik. These houses are again led by community members who wish to offer contextually and culturally relevant care to families.

In discussion with these committees, what seems to support them is the creation of times and spaces where Inuit can work together so that they can share and validate their common experiences, and create a common lexicon to speak about this knowledge in in their language (Inuktitut). This process can be referred to as the Indigenization of knowledge [27, 28]. Committees generally wish to develop common objectives and then be heard at decision making tables. Together they rethink the norms that structure the interactions between colleagues and between workers and clients. It takes time, trial and error, and most of all, it requires being heard [29]. To do so such initiatives need stable funding for the plans and projects already being developed by Inuit in order for them to have the time and resources to create and put in place their action plans.

## Conclusion

We looked specifically at Northern Quebec, however, the lessons learned are far from unique to this region.

Systemic racism refers in part to the structural realities that impact upon our collective ability to integrate a diversity of knowledges and ways of doing within our systems of care, impacting our ability to offer quality and culturally safe care to a diversity of peoples. It cannot be dismantled by simply sanctioning racism. Dismantling systemic racism requires long-term transformations of the policies, funding opportunities and educational practices that forge our systems of care. It means critically exploring the inequalities and the reasons behind such inequalities. It is the collective work of professional orders, universities, ministries and more, to observe what communities and the Inuit institutions are doing and find innovative strategies to support these initiatives without unduly placing pressure on those who are actively transforming the systems of care.

## Data Availability

All data and materials referred to in this paper and related to the first authors’ research is in the possession of the first author and can be available upon request to the Nunavik Board of Health and Social Services or to the Tasiurvik Family House.
